# Assessing fear learning via conditioned respiratory amplitude responses

**DOI:** 10.1111/psyp.12778

**Published:** 2016-12-08

**Authors:** Giuseppe Castegnetti, Athina Tzovara, Matthias Staib, Samuel Gerster, Dominik R. Bach

**Affiliations:** ^1^ Department of Psychiatry, Psychotherapy, and Psychosomatics University of Zurich Zurich Switzerland; ^2^ Neuroscience Centre Zurich University of Zurich Zurich Switzerland; ^3^ Wellcome Trust Centre for Neuroimaging University College London London UK

**Keywords:** Psychophysiological model, Fear conditioning, Respiration, Heart period, General linear model

## Abstract

Respiratory physiology is influenced by cognitive processes. It has been suggested that some cognitive states may be inferred from respiration amplitude responses (RAR) after external events. Here, we investigate whether RAR allow assessment of fear memory in cued fear conditioning, an experimental model of aversive learning. To this end, we built on a previously developed psychophysiological model (PsPM) of RAR, which regards interpolated RAR time series as the output of a linear time invariant system. We first establish that average RAR after CS+ and CS− are different. We then develop the response function of fear‐conditioned RAR, to be used in our PsPM. This PsPM is inverted to yield estimates of cognitive input into the respiratory system. We analyze five validation experiments involving fear acquisition and retention, delay and trace conditioning, short and medium CS‐US intervals, and data acquired with bellows and MRI‐compatible pressure chest belts. In all experiments, CS+ and CS− are distinguished by their estimated cognitive inputs, and the sensitivity of this distinction is higher for model‐based estimates than for peak scoring of RAR. Comparing these data with skin conductance responses (SCR) and heart period responses (HPR), we find that, on average, RAR performs similar to SCR in distinguishing CS+ and CS−, but is less sensitive than HPR. Overall, our work provides a novel and robust tool to investigate fear memory in humans that may allow wide and straightforward application to diverse experimental contexts.

Cued fear conditioning is an experimental paradigm commonly employed in the study of animal and human aversive memory. It relies on successfully learning the association between a neutral precursor (conditioned stimulus, CS) and an aversive sensory stimulation (unconditioned stimulus, US). While CS+ in rodents often elicit overt reactions (e.g., freezing), this is not the case in humans. Instead, the assessment of fear learning relies on measuring the activity of the autonomic nervous system, for example, via skin conductance responses (SCR, Boucsein, 2012), cardiac responses (Bohlin & Kjellberg, 1978; Castegnetti et al., 2016; Headrick & Graham, 1969; Obrist, Webb, & Sutterer, 1969), pupil size responses (Kluge et al., 2011; Korn, Staib, Tzovara, Castegnetti, & Bach, 2016), or on assessing a modulation of the startle reflex (Brown, Kalish, & Farber, 1951; Khemka, Tzovara, Gerster, Quednow, & Bach, 2016).

Whether respiratory physiology could also be informative about fear memory is less well known. Instructed threat results in altered respiration period and amplitude on a time scale of minutes (Svebak, 1982; Willer, 1975). Actual aversive events influence respiratory period, amplitude, and flow rate, on a time scale of seconds (Bach, Gerster, Tzovara, & Castegnetti, 2016). Moreover, one study suggested changes in respiratory period and, relatedly, end‐tidal carbon dioxide pressure (PetCO_2_), as a result of aversive conditioning (Van Diest, Bradley, Guerra, Van den Bergh, & Lang, 2009). However, we have recently shown that respiration amplitude responses (RAR) may be better suited to distinguish cognitive processes than respiration period (Bach et al., 2016). This motivates our current study in which we consider a possibility that conditioned changes in RAR may allow assessing fear memory. Respiration amplitude relates to tidal volume but, importantly, our aim is not to precisely measure tidal volume, for which a double‐belt system would be required to simultaneously capture both thoracic and abdominal compartments (Binks, Banzett, & Duvivier, 2007). However, if the ratio between thoracic and abdominal contribution is relatively constant within any individual, it may be possible to approximate tidal volume up to a linear constant from a single chest belt system. Here, we rely on the type of single belt system standardly used in MRI scanners for correcting breathing artifacts (Glover, Li, & Ress, 2000; Hutton et al., 2011). Our method could thus be easily applicable to a large number of existing experimental setups.

We capitalize on a previously developed psychophysiological model (PsPM) of event‐related RAR (Bach et al., 2016), which is formalized as a general linear model (GLM). We first analyze RAR to CS+ and CS− to establish an impulse response function for fear‐conditioned RAR. This PsPM is then inverted to yield an amplitude estimate of the CS‐associated input into the respiratory system, which is assumed to directly relate to fear memory. We then compare this model to peak‐scoring methods. A priori, the advantage of a model‐based approach is that it circumvents the choice of a pre‐event baseline time window. In the case of short intertrial intervals (ITI), this choice could potentially bias peak‐scoring estimates. The GLM implementation, instead, estimates the baseline from the entire experimental time course, thus providing a principled approach that allows meaningful comparison across studies. Second, the PsPM embodies assumptions about what response components are relevant in distinguishing between conditions and what can be treated as noise. Indeed, this approach has been shown to afford more precise distinction of CS+/CS− trials (i.e., predictive validity) in the analysis of SCR (Bach, Flandin, Friston, & Dolan, 2010; Staib, Castegnetti, & Bach, 2015), heart period responses (HPR, Castegnetti et al., 2016), and pupil size responses (Korn et al., 2016). To ensure that our results are not driven by features of the particular data used for model construction, we validated the method on five further data sets. These validation data sets involve different experimental designs and two different types of chest belts to trace the respiratory activity, namely a bellows and a pressure cushion system, to provide for wide applicability.

## Method

### Participants

Five independent samples of healthy, nonmedicated individuals were recruited from the general population; for one of these samples, we analyze both an acquisition and an extinction data set. All participants confirmed that they had no history of neurological, psychiatric, or systemic disorders, and all had normal or corrected‐to‐normal vision. We recorded data from 35 (Experiment 1, 23 females, aged 18–31 years, 23.4 ± 3.4), 23 (Experiment 2, 10 females, aged 20–32 years, 23.8 ± 3.0), 23 (Experiment 3, 13 females, aged 19–33 years, 26.2 ± 4.8), 21 (Experiment 4, 8 females, aged 19–34 years, 25.7 ± 4.6), and 20 (Experiment 5, 12 females, aged 18–30 years, 22.8 ± 3.3) participants, for the five experiments. Because of electrode detachment or malfunctioning of the data recording equipment, we excluded two subjects from Experiment 1 and 5, four from Experiment 2, three from Experiment 3, and five from Experiment 4. Three further subjects were excluded from Experiment 1 because they stated after the experiment to not have perceived the US. One participant did not complete Experiment 1. Therefore, for the five experiments, 29, 19, 20, 16, and 18 subjects, respectively, were included in the data analysis. All participants gave informed written consent before the experiment. The study was conducted in accordance with the Declaration of Helsinki and approved by the governmental research ethics committee (Kantonale Ethikkommission Zürich). HPR and SCR data from Experiment 1, 2, and 4 were included in a previous methodological report (Castegnetti et al., 2016).

### Experimental Procedure

#### Common settings

Unconditioned stimuli (US) were trains of electric square pulses delivered on participants' dominant forearm with a pin‐cathode/ring‐anode configuration. The stimulus intensity was set such that the perceived intensity was at around 90% of the pain threshold. We estimated the pain threshold in two phases. First, the intensity was gradually increased from being unperceivable to a painful level. This determined an upper threshold for the second phase, in which the subjects were asked to rate the perceived intensity of 14 stimuli with different intensities. These ratings were then linearly interpolated to estimate the intensity corresponding to 90% of the pain threshold. In the MRI Experiment 4, visual stimuli were presented via MR‐compatible goggles with a resolution of 800 × 600 pixels (Resonance Technology Inc., Northridge, CA). For all the other experiments, we used a 20″ diagonal LCD screen with an aspect ratio of 16:9 and a resolution of 1,280 × 1,024 pixels at 50 Hz (P2014HT, Dell, Round Rock, TX). With the exception of Experiment 5 (see dedicated paragraph for details), the duration of the ITI was randomly determined to be 7, 9, or 11 s.

#### Experiment 1

Experiment 1 (data set code: FR) consisted of an acquisition and an extinction/retention session. For the acquisition, we used a delay fear conditioning paradigm with visual CS. The US was a series of 250 1‐ms long, square electric pulses delivered at a frequency of 500 Hz, resulting in a total US duration of 0.5 s. Current intensities were set to values between 1.0 and 6.7 mA (mean ± *SD*, 2.6 ± 1.28 mA). Participants were exposed to 160 CS: 80 CS+, half of which coterminated with the US, and 80 CS− that predicted the absence of the US. The two CS were two different colors (screen plain blue or red for CS+/CS−) on a computer screen. The US was delivered 3.5 s after the CS onset; CS and US coterminated 0.5 s later. Participants were instructed to report the color on the screen by pressing one of two designated buttons on the keyboard. Both the CS+/CS− colors and the button associations were counterbalanced across subjects. During the extinction/retention session, participants were told to expect a shorter (six CS+ and six CS− only), but otherwise identical, experimental paradigm as the one employed in the acquisition phase. The electrodes were again placed on participants' forearm, but no shock was delivered. In this session, 16 participants were additionally presented a startling auditory stimulation 3.8 s after the CS onset, which has been shown to elicit respiratory responses by itself (Bach et al., 2016) and thus interfere with the CS responses. Thus, only data from the remaining 13 participants were included in the analysis of the extinction/retention session.

#### Experiment 2

This was a trace fear conditioning task (data set code: TC) with the same CS and US as in Experiment 1. Currents were between 1.0 and 7.0 mA (mean ± *SD*, 3.0 ± 1.3 mA). CS were presented for 3 s, after which a fixation cross appeared, followed 1 s later by the US in 50% of the CS+ trials.

#### Experiment 3

The design of Experiment 3 (data set code: DoxMemP) was similar to Experiment 1. The acquisition session was identical to Experiment 1, while the extinction session contained startle sounds for all trials and subjects, and was thus not analyzed in the present report. Individual electric current intensities were between 1.25 and 8.60 mA (mean ± *SD*, 2.82 ± 1.65 mA).

#### Experiment 4

Experiment 4 (data set code: VC1F) was a delay fear conditioning task with visual CS, performed during MRI scanning. It consisted of 16 blocks of 12 trials each. Eight blocks entailed only explicitly instructed nonreinforced trials, and are not analyzed here. The remaining eight blocks contained overall 96 trials, evenly divided into CS− and CS+. Half of the CS+ coterminated with the US. US consisted of five square electric pulses with 0.2‐ms duration and delivered at a frequency of 10 Hz, resulting in a total stimulus duration of 0.5 s. Current intensities were between 6 and 45 mA (mean ± *SD*, 17.2 ± 12.2 mA). Two pairs of CS were presented, either simple (during four blocks) or complex (during the other four blocks). Simple CS were Gabor patches with different orientation (290° or 340°, counterbalanced across participants), while complex stimuli consisted of simple stimuli overlaid with an additional Gabor patch oriented at 230°. Both simple and complex CS lasted 4 s. A 2 × 2 analysis of variance (ANOVA) between complexity and CS type revealed no main effect of complexity and no interaction between complexity and CS type. Therefore, as indexed by SCR, simple and complex CS appeared to elicit similar fear learning; they were then collapsed for analysis of RAR.

#### Experiment 5

Experiment 5 (data set code: LI) was a delay fear conditioning paradigm with auditory CS and longer stimulus onset asynchrony (SOA). Eighty CS+ and 80 CS− were presented to each participant, and consisted of sine tones with constant frequency (220 and 440 Hz), delivered via a headset for 6.5 s, with intensity of about 80 dB. Electric shocks with a duration of 0.5 s served as US, and occurred 6 s after 50% of the CS+ onsets, with electric currents between 1.0 and 7.0 mA (mean ± *SD*, 3.2 ± 1.3 mA). The ITI was randomly determined to be 11, 15, or 17 s. Participants were instructed to report the type of the CS by pressing one of two designated keys on the keyboard. Both the tone‐key and the tone‐CS associations were counterbalanced across subjects.

#### Psychophysiological recording

In Experiment 1, 2, 3, and 5, respiratory time series were collected with an aneroid chest bellows (V94‐19, Coulbourn Instruments, Whitehall, PA) and differential aneroid pressure transducer (V94‐15, Coulbourn). The signal was amplified with a resistive bridge strain gauge transducer coupler (V72‐25B Coulbourn). We simultaneously recorded the electrocardiogram with four 45‐mm, pregelled Ag/AgCl adhesive electrodes attached to the four limbs. The experimenter visually identified the lead (I, II, III) or the augmented lead (aVR, aVL, aVF) configuration that displayed the highest R spike, and only recorded this configuration. Data were preamplified and 50 Hz notch‐filtered with a Coulbourn isolated five‐lead amplifier (LabLinc V75‐11, Coulbourn Instruments). Skin conductance was recorded from the thenar/hypothenar of the nondominant hand using two 8‐mm disk Ag/AgCl cup electrodes (EL258, Biopac Systems Inc., Goleta, CA) and 0.5% NaCl gel (GEL101, Biopac Systems Inc.; Hygge & Hugdahl, 1985) and fed into an SCR coupler/amplifier (V71‐23, Coulbourn Instruments). All the data time series were digitized at 1000 Hz using a Dataq card (DI‐149, Dataq Inc., Akron, OH) and recorded with Windaq (Dataq Inc.) software. SCR data from Experiment 5 were not analyzed as it appears that the best way of modeling SCR may depend on the SOA, and a suboptimal model could give the respiration measure an unfair advantage.

In Experiment 4, respiratory time series were recorded, at a sampling rate of 496 Hz, with an MRI‐compatible pressure cushion system. Cardiac activity was measured at 500 Hz via a peripheral pulse oximeter (PPO, SpO2 adult grip, Invivo, Gainesville, FL) placed around the nondominant index finger and connected to a wireless peripheral pulse unit via fiber optic. Both the respiratory and the cardiac activity signals were transmitted to a wireless triggering unit and then to the MRI console for recording. Skin conductance was recorded with a data acquisition system (MP150, Biopac Systems Inc.) coupled to a signal amplifier (GSR‐100C, Biopac Systems Inc.) at 1000 Hz sampling frequency.

### Data Preprocessing

Data processing and analysis were performed with MATLAB (Version R2015a, MathWorks Inc., Natick, MA), using routines in PsPM 3.0 and custom‐written code. Raw respiratory traces were converted to interpolated respiration amplitude time series with a previously published respiratory cycle detection algorithm (Bach et al., 2016). The algorithm detected the sharp pressure reduction recorded with chest bellows systems during inspiration. In particular, respiratory time series were mean centered, band‐pass filtered (0.01–0.6 Hz), and median filtered over 1 s. The resulting negative zero crossings were set as the start of the inspirations. The method was validated in the context of our previous report by comparison with the visual detection of respiratory cycles by a trained expert (SG) on a data set not used for developing the algorithm. As a result, the automatic procedure had a sensitivity of 99.3% and true predictive validity of 99.5%. For data obtained from the cushion/belt system, the onsets of the inspirations were defined as the minima of the respiratory trace. Hence, to analyze data from Experiment 4, the algorithm was adapted to extract zero crossings of the derivative of the time series (i.e., the extrema), from which the positive ones (i.e., the minima) were set as the start of the inspiration. A modified offline implementation (Paulus, Castegnetti, & Bach, 2016) of the Pan and Tompkins (1985) real‐time QRS detection algorithm was used to identify QRS complexes, and translated into a heart period time series. To extract the heart beats from the PPO time series in Experiment 4, we used a custom template‐matching algorithm as reported previously (Castegnetti et al., 2016). Respiration amplitude time series were band‐pass filtered with a bidirectional Butterworth filter, with low‐pass and high‐pass cutoffs of 2 Hz and 0.01 Hz, respectively. These were chosen to remove high‐frequency noise and the effects of possible slow movements of the recording device during the experimental session. Single‐trial responses were analyzed in a 11‐s time window starting from the CS onset, corresponding to the minimum time interval between subsequent CS onsets. For model development and model‐free scoring techniques, single‐trial responses were baseline‐corrected by subtracting the respiratory amplitude averaged during the 4 s before the CS onset, in line with previous approaches to the cardiac response (Pollatos, Herbert, Matthias, & Schandry, 2007). This baseline window reconciles the need to average over approximately an entire respiratory cycle in the respiration amplitude, and to minimize the effect of the previous trial. Heart period time series were band‐pass filtered with a bidirectional Butterworth filter (0.015–0.5 Hz) as previously established (Castegnetti et al., 2016). SCR data were filtered with a bidirectional Butterworth band‐pass filter (0.0159–5 Hz), as recommended for analysis of fear‐conditioned SCR (Staib et al., 2015). For statistical analysis of the HPR, we used a general linear convolution model with a heart period response function derived from Experiment 1 (Castegnetti et al., 2016). For SCR analysis, we used the default nonlinear model with a conditioned response window during the entire CS/US interval, as implemented in PsPM 3.0 (http://pspm.sourceforge.net, Bach, Daunizeau, Friston, & Dolan, 2010; Staib et al. 2015).

### Model Specification

In agreement with a previous approach (Bach et al., 2016), we modeled the interpolated RAR as output of a linear time invariant system (LTI) that receives a brief impulse input upon detection of a CS. LTI systems have two characteristic properties: first, the output does not explicitly depend on time (time invariance); second, the response to several inputs is the sum of the responses to the single inputs (linearity). In most real systems, including the respiratory system, these criteria are not strictly met. Indeed, the assumption of pure summation of overlapping inputs is unrealistic when studying the amplitude of the respiratory cycle, which is ultimately bounded by functional residual capacity and total lung capacity. However, we assume that, with our choice of the ITI, the approximation is accurate enough for the LTI formalism to be applicable. Thus, if an input x(t) produces the output  y(t), then the input x(t+δ), with δ∈R, elicits y(t+δ). An LTI system is fully specified by its response function (RF). Since the operation of convolution between the functions *f* and *g* is defined as

$$\left(f*g\right)\left(t\right)=\int_{0}^{\infty }f\left(\tau \right)g\left(t-\tau \right)d\tau ,$$

we can then model the response of a LTI system to an input by convolving this input with the RF, i.e.,

$$y\left(t\right)=RF\left(t\right)*x\left(t\right).$$

Here, we assume an impulse input at CS onset, meaning that our definition of RF should be interpreted as summarizing neural and peripheral processes translating the external stimulus into a respiratory response. Therefore, since the combination of these processes is not accessible to the experimenter, we phenomenologically construct the RF by studying the response of the system to a set of known inputs. This approach led to the successful development of a model for SCR (Bach, Flandin, Friston, & Dolan, 2009; Bach, Flandin et al., 2010), HPR (Castegnetti et al., 2016), pupil (Korn et al., 2016), and startle responses (Khemka et al., 2016).

#### GLM

After defining a forward model that specifies how a cognitive input produces RAR, we need to invert this model to infer the most likely input given the observed data. If the input onset is known, we can estimate its amplitude using a GLM. Specifically, if *Y* is a set of *k* observation and *X* is a design matrix, we can write a GLM as

Y=Xβ+ϵ,

where *ϵ* is independent and identically distributed noise. In our case, the columns of the design matrix *X*, one per experimental condition, contain an impulse input for each presentation of the condition, separately convolved with the single components of the RF. Hence, in the above formula, the vector of regression weights β corresponds to the amplitude of the inputs. To infer the most likely amplitude estimates $\hat{\beta },$ we calculated the Moore‐Penrose pseudoinverse $X^{+}$ with the maximum likelihood method implemented in the MATLAB function *pinv*:

$$\hat{\beta }=X^{+}Y.$$

#### Model construction

We built a response function aimed at discriminating between the RAR to CS+ and CS−. To this end, we built the RF from the difference between the grand means of the responses to the two different stimuli obtained from the first data set, as in a previous approach to HPR (Castegnetti et al., 2016) and pupil responses (Korn et al., 2016). As a first step, we visually identified a function class that qualitatively resembled the shape of the difference between grand means. A gamma distribution seemed a suitable candidate. To formalize the RF, we used the Nelder‐Mead algorithm implemented in the MATLAB function *fminsearch* (Lagarias, Reeds, Wright, & Wright, 1998) to find the values of the shape parameter *k*, the scale parameter *ϴ*, and the time onset *x_0_* that minimized the residual sum of squares (RSS) from the gamma function

$$y=\frac{A}{\theta^{k}\Gamma (k)}{\left(x-x_{0}\right)}^{k-1}e^{-\frac{x-x_{0}}{\theta }}.$$

**Figure 1:**
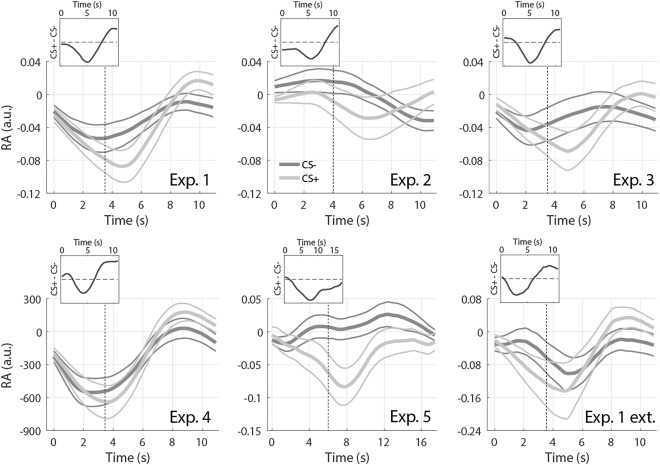
Respiratory amplitude response. Response to CS+ (light gray) and CS‐ (dark gray), averaged across participants and trials, ± *SEM* (thin lines), obtained from the six data sets. Vertical dashed lines indicate US onset. Insets: Difference between average RAR to CS+ and CS‐.

**Figure 2:**
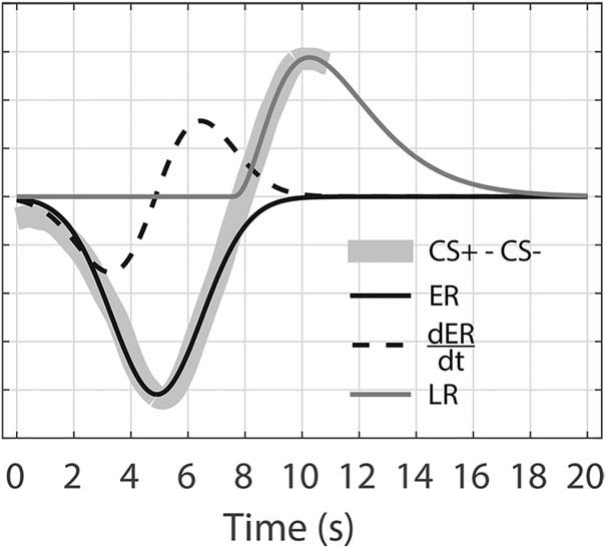
Response functions. Early (ER) and late (LR) RFs obtained from the difference between the RAR from Experiment 1.

We term this the early respiration amplitude response function (ER) and formalize it as model G1 (Figure 1). To allow for subject‐specific variations in peak latency, we included its time derivative ( $\frac{dER}{dt}$ in Figure 1) as a second component in model G2, analogous to previous approaches to fMRI, SCR, and RAR (Bach et al., 2009, 2016; Friston et al., 1998). Finally, we observed that the RAR differentiates between conditions also at a later stage, namely, in a window between 8 s and the end of the considered response (11 s). We therefore created a third model, G3, which comprises the ER and a late response function (LR, Figure 2). Finally, we also defined a model G4 that includes ER, its derivative, and LR. To estimate autonomic input from models G1 and G2, we reconstructed the estimated RAR from the entire basis set and calculated the signed maximal variation from baseline of this reconstructed response between 2 and 11 s after CS onset, as established previously for SCR (Bach, Friston, & Dolan, 2013) and HPR (Castegnetti et al., 2016). In contrast, models G3 and G4 scored RAR in terms of the signed difference between the estimated amplitudes of LR and ER.

#### Model comparison and validation

Together with the model‐based approaches presented above, we tested a number of model‐free methods. In particular, we scored the RAR (a) by the amplitude of the maximum positive peak in a time window between 2 and 11 s after the CS onset, (b) by the signed amplitude of their maximal variation from baseline in this window, and (c) by the average RAR within a window of 2–7 s. The interval for method (c) is shorter because the discrimination power of the area under the curve crucially depends on the width of the curve. We thus optimize this width on the first data set, analogous with the model‐based approaches. To quantify predictive validity, we calculated evidence for a model in which CS+ and CS− estimates are drawn from distributions with the same variance but different means. We did this by calculating the RSS from a regression model in which the vector of event types is the dependent variable, and the estimated responses per condition are the predictor, and that contained subject‐specific intercept terms, analogous to a repeated measures ANOVA or paired *t* test. This RSS can be transformed into the Akaike information criterion (AIC) via (Burnham & Anderson, 2004)

$$AIC=n\;\log\left(\frac{1}{n}RSS\right)+2k,$$

with *n* number of observations and *k* parameters, which was the same for all models and therefore drops from AIC differences. A smaller AIC indicates a higher model evidence. An absolute AIC difference higher than 3 is often regarded as decisive, by analogy to a classic *p* value (Penny, Stephan, Mechelli, & Friston, 2004; Raftery, 1996). Hence, the main advantage of calculating the AIC instead of *t* or *p* values is that AIC allows a meaningful comparison between models.

To investigate the effects of the CS‐US SOA on the RAR, Experiment 2 and 5 employed different SOAs (4 and 6 s, respectively, instead of 3.5 s as in Experiment 1, 3, 4, and 5). We tested two model adjustments on data set 5, which had longer SOA and therefore allows better disambiguation between these possibilities: (a) the RF is time‐locked to the CS and is thus unaffected by the SOA, and (b) the RF is unchanged in shape and time‐locked to the US (model G1‐4′). We then validated the winning adjustment on data set 2.

Although there was no reason to assume that the presented models were biased toward any experimental condition, we nonetheless sought to empirically assess the unbiased nature of the model‐based approach. We did this on data set 1 by randomly permuting the trial indices, thus creating sets of RAR in which both conditions contained CS+ and CS− trials and responses in these conditions are therefore not expected to differ. This permutation was repeated 1,000 times and results averaged.

## Results

We first ensured that participants learned the contingency between CS and US. To do this, we contrasted the response to CS+ and CS− in two established psychophysiological measures: SCR and HPR. As a result, we found that SCR and HPR significantly discriminated CS+/CS− in all experiments (Table 1).

**Table 1:** SCR and HPR Results

Experiment		SCR				HPR			
	*df*	*|t|*	*p*	*d*	AIC	*|t|*	*p*	*d*	AIC
1	28	3.23	.0032	0.60	−98.8	5.85	<.001	1.09	−126.7
2	18	3.58	.0021	0.82	−73.1	4.97	<.001	1.14	−85.2
3	19	3.46	.0026	0.77	−68.4	4.67	<.001	1.04	−86.0
4	15	2.6	.022	0.65	−56.0	2.12	.051	0.53	−52.8
5	17	n.a.	n.a.	n.a.	n.a.	3.83	.0013	0.90	−72.4
1 retention	12	2.17	.047	0.60	−50.3	2.33	.038	0.65	−45.7

*Note*. The *t* and the *p* values are obtained from a paired *t*‐test between subject‐averaged responses to CS+ and CS‐, together with the ensuing predictive validity in terms of Akaike information criterion (AIC) and effect size *d*. *n.a*. = not available.

### Respiratory Amplitude Response Function

The grand means of the RAR to CS+ and CS−, obtained from the six data sets, are shown in Figure 1. With the exception of Experiment 2 and 5, which involved different CS‐US timings, respiratory amplitude after CS+ tends to be lower than after CS− in a window centered around 4–5 s after the CS onset. A respiratory amplitude higher after CS+ was observed about 8–11 s from the CS onset. In addition, we found that the two traces typically start to diverge about 2 s after the CS onset (i.e., 1.5 s before the US), ruling out the possibility that the observed differences were only due to expected or unexpected US omission at 3.5 s after CS onset. Response functions were built on data from Experiment 1 exclusively, and were obtained by subtracting the average RAR to CS− from CS+, analogous with a previous approach (Castegnetti et al., 2016). As stated above, we observed two critical windows in which RAR seemed to discriminate the conditions. With this in mind, we sought to construct separate components of the RF for each of these windows. These components were later combined to obtain different model variants. The gamma distribution that best fitted the earlier difference had parameters k = 2.57010 · 10^7^, ϴ = 3.12410 · 10^−4^, x_0_ = −8.02434 · 10^3^. The value of the fitted amplitude A was left as a free parameter during the GLM implementation. The late RF was obtained in the same way, but restricting the fit to the 8–11 s window, resulting in parameters k = 3.41302, ϴ = 1.10734, x_0_ = 7.58288. The difference between the RAR to CS+/CS−, together with the fitted early and late RFs, are depicted in Figure 2.

### Model Comparison

Next, we compared the different models in terms of their predictive validity, expressed as AIC. Model G3 (ER + LR) best discriminated CS+/CS−, but model G2 (ER + $\frac{dER}{dt}$) was not decisively worse (AIC_G3_ − AIC_G2_ = 1.8, Table 2). G2 and G3 were thus selected as best models and used for model validation.

**Table 2:** Model Comparison

#	Model description	AIC	*|t|*	*p*
P1	Maximum variation from baseline	−80.7	0.40	.69
P2	Peak scoring	−82.3	0.96	.35
P3	Average in the 2–7 s window	−87.7	1.94	.063
G1	Canonical response	−98.6	3.21	.0033
G2	ER + $\frac{dER}{dt}$	−100.2	3.37	.0022
G3	ER + LR	−102.0	3.56	.0014
G4	ER + $\frac{dER}{dt}$ + LR	−93.5	2.67	.013

*Note*. Akaike information criterion (AIC, smaller is better) together with the *t* and the *p* values obtained from model‐free (P1 to P3) and the model‐based (G1 to G4) evaluations of the RAR in Experiment 1 (development data set). Absolute AIC differences larger than 3 are considered decisive. Models G2 and G3 outperform all alternative methods.

### Model Validation

**Figure 3:**
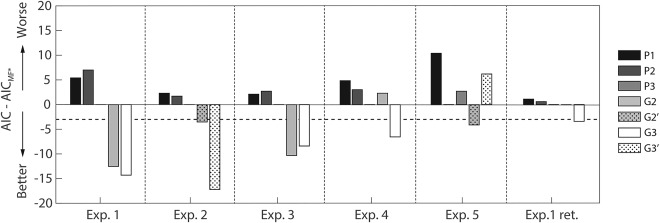
Model comparison. Bars represent predictive validity in terms of AIC (smaller is better) for three model‐free methods (P1–P3) and the winning models G2 and G3. For each experiment, we plotted the AIC with respect to the best model‐free method MF* (P1 for Experiment 4; P2 for Experiment 5; P3 for Experiment 1, 2, 3, and 1 extension). The horizontal dashed line represents the threshold below which AIC values are decisively better than MF*. For models G2 and G3, effect sizes (Cohen's *d*) were, respectively: 0.63, 0.66 (Experiment 1); 0.40, 0.83 (Experiment 2); 0.64, 0.59 (Experiment 3); 0.29, 0.64 (Experiment 4); 0.71, 0.38 (Experiment 5); 0.20, 0.42 (Experiment 1 retention).

Up to this point, we had compared models on the same data set on which the response functions were developed, which is susceptible to overfitting. We therefore validated our results on five independent data sets. The results are displayed in Figure 3. In all short SOA experiments, G3 had higher predictive validity than any peak‐scoring method. G2 performed similar to G3 in Experiment 1 and 3 and similar to peak scoring in Experiment 4 and in the fear retention phase of Experiment 1.

To validate the model on data sets 2 and 5, involving a longer SOA, we first sought to identify the best way to adapt the model to different SOAs. We did this on data from Experiment 5, with the result that the CS− and US‐locked variants G2 (AIC_*G2*_ = −65.4) and G2′ (AIC_*G2′*_ = −65.4) did not differ from each other, and both outperformed peak scoring. In contrast, the two variants of G3 were not better than peak scoring, but G3′ was better than G3. On average, shifting the RF with the US thus appears to be the better of the two tested adaptations to different SOAs. When we tested it on data set 2, however, the performance of the US‐locked G2′/3′ was similar to their CS‐locked counterparts G2/G3. This may follow from the fact that the SOA in Experiment 2 is only 0.5 s longer, causing G2′/3′ to differ slightly from G2/3, and possibly not enough to produce substantially different results. In Figure 3, we show model evidence for G2′ and G3′.

Finally, although we had no theoretical reason to believe that our model‐based approach overestimated CS+/CS− differences, we sought to rule this out empirically. We assessed this by analyzing RAR in two conditions that do not systematically differ. We randomly assigned CS+ and CS− trials to either condition. We performed this analysis on data set 1, finding that neither G2 nor G3 differentiates between conditions (G2: *t*(28) = −0.03, *p* = .50; G3: *t*(28) = 0.22, *p* = .48), as expected.

### Comparison with Skin Conductance and Heart Period Responses

**Figure 4:**
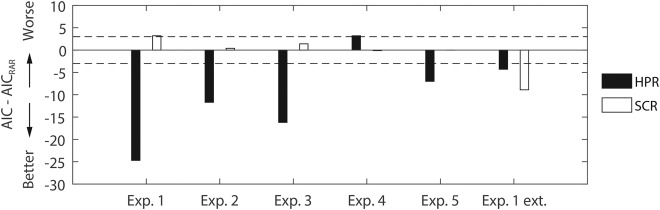
Comparison of predictive validity (AIC, smaller is better) between RAR, HPR, and SCR. The horizontal dashed lines represent the decision thresholds with respect to RAR. Positive and negative values indicate performances lower and higher than those obtained from RAR, respectively. For Experiment 5, SCR analysis was not available. For Experiment 2 and 5, HPR analysis was adapted to the longer trace between CS and US by time‐locking the heart period response function to the US onset, similar to the RAR analysis.

To relate the above results to other psychophysiological measures of fear learning, we compared the performance achieved by the best model‐based method for analyzing RAR to the performance of analogous methods for the analysis of HPR and SCR. The results are depicted in Figure 4. In the development data set 1, the RAR‐based analysis performed better than SCR, but worse than HPR; although as this data set was used to develop the model‐based approaches for RAR and, in a previous report, for HPR, this comparison may be biased against SCR. In the validation data sets, we observed a more nuanced pattern. RAR was comparable to SCR in Experiment 2, 3, and 4; no SCR results were available for Experiment 5. During fear retention under extinction (Experiment 1 retention), SCR performed better than RAR. HPR was markedly better than RAR in all data sets, except fMRI Experiment 4, in which RAR won over HPR. Note that, for the long SOA Experiment 5, we used a US‐locked version of the heart period response function for the HPR analysis, as in our previous study. To support this choice, we compared evidence for a CS− and a US‐locked HPR model, with the result that the US‐locked model (AIC = −72.4) outperformed the CS‐locked version (AIC = −56.4), just as in our previous investigation with a 4‐s SOA.

**Figure 5:**
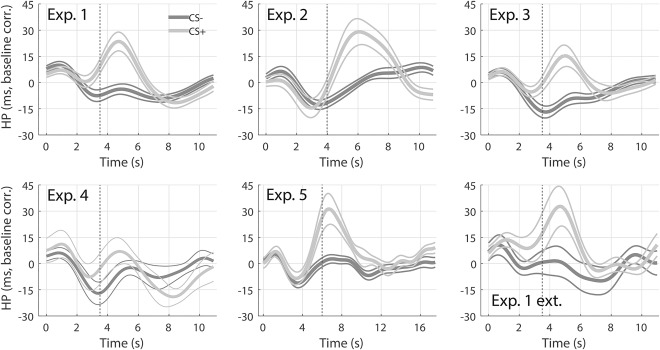
Heart period response. Response to CS+ (light gray) and CS‐ (dark gray), averaged across participants and trials, ± *SEM* (thin lines), of the six data sets in Figure 1. Vertical dashed lines indicate the US onset.

Finally, to put the present results into a physiological perspective, we qualitatively compared average RAR (Figure 1) with simultaneously recorded HPR (Figure 5). Remarkably, to some extent, the two measures appear to inversely correlate: longer heart periods appear to be associated with lower respiratory amplitudes. This is in line with the known effect of respiration on cardiac activity, termed respiratory sinus arrhythmia, that causes increases in the heart rate during periods of higher lung volume (Yasuma & Hayano, 2004), and suggests that, through its effect on heart period, RAR to CS+ may contribute to the observed HPR over and above direct parasympathetic control of fear‐conditioned bradycardia.

## Discussion

In this paper, we present a novel technique for assessing human fear learning from respiration recordings. First, we show differential RAR to CS+ and CS−. We then use average RAR to formulate a response function for a PsPM. This PsPM is inverted to yield estimates of cognitive input amplitudes. These estimates are compared to model‐free methods commonly used in psychophysiological research (e.g., peak‐scoring, Furedy & Poulos, 1976) in terms of their predictive validity, that is, the ability to distinguish CS+ and CS−. It turns out that the PsPM outperforms all model‐free methods, both in the development data set and in five validation data sets.

In particular, model G3, consisting of an early and a late component, was the best model for experiments with short CS‐US SOA (3.5–4 s). Only in long SOA (6 s) Experiment 5, model G2 with early response and its derivative, won the model comparison. This reflects the descriptive observation that responses in Experiment 5 lack the late positive component, which, in contrast, is observed in all other data sets (Figure 1, insets). However, the origin of this discrepancy is unclear: besides the longer SOA, this was also the only experiment using auditory CS instead of visual CS. Furthermore, we were interested whether ER and LR could relate to RAR elicited by short external events previously reported (Bach et al., 2016). With a peak latency of 8.1 s, previously observed RAR could relate to LR, which peaks at a longer latency (10.2 s), but not to the short latency (5.1 s) ER. This is different from skin conductance (Bach, Daunizeau et al., 2010), heart period (Castegnetti et al., 2016), or pupil size (Korn et al., 2016) responses, for which such relation between evoked and fear‐conditioned responses could be made. We note that the LTI system assumed to control RAR probably collapses a broader range of neural and peripheral components than the models associated with other psychophysiological measures.

To put our results into a psychophysiological context, we compared predictive validity of RAR to HPR and SCR. On average, RAR performance turned out to be comparable to SCR, but worse than HPR. However, due to large trial‐by‐trial variability, RAR and HPR analysis is implemented conditionwise, while the SCR is analyzed on a trial‐by‐trial basis. It thus allows analyzing of the evolution of the physiological response during the time course of a single experimental session, giving SCR an additional advantage over RAR.

Finally, for each data set, we compared the average respiratory amplitude (Figure 1) to heart period (Figure 5). Qualitatively, the plots suggest an inverse relationship between the two, since onsets of the positive peak of the HPR and of the negative peak of the RAR appear to correlate. This does not surprise, though, as lung volume and cardiac frequency are known to be related (i.e., respiratory sinus arrhythmia, Yasuma & Hayano, 2004). Although it is difficult to conclude the direction of causality, an intriguing hypothesis is that the parasympathetic response to CS+ has a dual effect on heart period: a direct one via cardiac afferents and an indirect one via the respiratory system. As a further speculation, this could possibly be the reason for the overall higher predictive validity of HPR compared to RAR. Additional investigation, possibly with tight control over central input into the heart, would be required to shed light on this question.

In summary, we introduce a novel, robust, model‐based approach for analyzing RAR in response to fear‐conditioned stimuli, which was tested during acquisition and extinction of fear learning. We show that the method generalizes to data sets with diverse experimental settings, conditioned stimuli, and types of breathing belts.
